# Inhibition of TRPM7 by carvacrol suppresses glioblastoma cell proliferation, migration and invasion

**DOI:** 10.18632/oncotarget.3872

**Published:** 2015-04-19

**Authors:** Wen-Liang Chen, Andrew Barszczyk, Ekaterina Turlova, Marielle Deurloo, Baosong Liu, Burton B. Yang, James T. Rutka, Zhong-Ping Feng, Hong-Shuo Sun

**Affiliations:** ^1^ Department of Surgery, University of Toronto, Toronto, Canada; ^2^ Department of Physiology, University of Toronto, Toronto, Canada; ^3^ Department of Pharmacology, University of Toronto, Toronto, Canada; ^4^ Laboratory Medicine and Pathobiology, University of Toronto, Toronto, Canada; ^5^ Institute of Medical Science, Faculty of Medicine, University of Toronto, Toronto, Canada

**Keywords:** glioblastoma, carvacrol, TRPM7, cell viability, migration, invasion

## Abstract

Glioblastomas are progressive brain tumors with devastating proliferative and invasive characteristics. Ion channels are the second largest target class for drug development. In this study, we investigated the effects of the TRPM7 inhibitor carvacrol on the viability, resistance to apoptosis, migration, and invasiveness of the human U87 glioblastoma cell line.

The expression levels of TRPM7 mRNA and protein in U87 cells were detected by RT-PCR, western blotting and immunofluorescence. TRPM7 currents were recorded using whole-cell patch-clamp techniques. An MTT assay was used to assess cell viability and proliferation. Wound healing and transwell experiments were used to evaluate cell migration and invasion. Protein levels of p-Akt/t-Akt, p-ERK1/2/t-ERK1/2, cleaved caspase-3, MMP-2 and phosphorylated cofilin were also detected.

TRPM7 mRNA and protein expression in U87 cells is higher than in normal human astrocytes. Whole-cell patch-clamp recording showed that carvacrol blocks recombinant TRPM7 current in HEK293 cells and endogenous TRPM7-like current in U87 cells. Carvacrol treatment reduced the viability, migration and invasion of U87 cells. Carvacrol also decreased MMP-2 protein expression and promoted the phosphorylation of cofilin. Furthermore, carvacrol inhibited the Ras/MEK/MAPK and PI3K/Akt signaling pathways.

Therefore, carvacrol may have therapeutic potential for the treatment of glioblastomas through its inhibition of TRPM7 channels.

## INTRODUCTION

GBM (Glioblastoma Multiforme) is a common primary brain tumor with aggressive proliferative and invasive characteristics; it has a 5-year survival rate of 9.8% in adults that undergo a combination of chemotherapy and radiotherapy [1]. The median overall survival is only 14.6 months after surgical resection, chemotherapy and radiation, which is due to the rapid proliferation and unrestricted migration of glioblastomas [1]. The potent ability of glioblastomas to migrate throughout brain tissue via the degradation of extracellular matrix is the main reason that surgery fails to completely remove glioblastomas from the brain [2]. Thus, the current standard treatment for GBM involves maximal surgical resection, followed by radiation and adjuvant chemotherapy using temozolomide [3]. Since, the effectiveness of GBM therapies is limited [4], the discovery of new and specific chemotherapeutic agents is welcomed.

Genetic abnormalities that enhance receptor tyrosine kinase (RTK)-mediated constitutive activation of Ras/MEK/MAPK and PI3K/Akt signaling pathways have been identified in human glioblastomas [5, 6]. It is for this reason that novel compounds targeting receptor tyrosine kinases (RTKs), vascular endothelial growth factor (VEGF) receptors, the PI3k/Akt/mTOR signaling pathway, and the MAPK signaling pathway are currently being evaluated in clinical trials [7].

The TRPM7 channel is a member of the melastatin subfamily within the transient receptor potential (TRP) ion channel superfamily. It is a divalent cation channel that conducts calcium, and it is ubiquitously expressed in almost all tissues. TRPM7 plays important role in anoxia/ischemia, development and cell proliferation [8-10]. Studies also show that ion channels are involved in the differentiation, proliferation, and invasion of tumors [11]. The specific involvement of TRPM7 has been demonstrated in the tumor cells of several cancer types, including breast cancer [12], gastric cancer, head and neck cancer, nasopharyngeal carcinoma, pancreatic cancer, prostate cancer, retinoblastoma and leukemia [13-17]. The role of TRPM7 in cancers is suggested by its up-regulation in the tissues of several cancer types [16, 18]. Therefore, TRPM7 can potentially serve as both a clinical biomarker and therapeutic target in a variety of cancers [19].

Several inhibitors of TRPM7 have been identified, including carvacrol [20], Waixenicin A [21], 2-aminoethoxydiphenyl borate (2-APB) [22], sphingosine and FTY720 [23]. Carvacrol is a naturally synthesized, bioactive monoterpenoid phenol with multiple uses. Its synthesis originates from the mevalonate pathway [24] and constitutes various proportions of oregano essential oils from numerous genera [25]. It's oral LD50 is 810 mg/kg in rats [26] and it is a “generally recognized as safe” food flavor additive according to the United States Food and Drug Administration. Carvacrol exhibits bioactivity in a variety of areas. It has antimicrobial, antibacterial, antiviral, antifungal and antiprotozoal properties thus suggesting its usefulness as an inhibitor of foodborne pathogens and human pathogenic bacteria [27]. In the physiological system, carvacrol exhibits anti-inflammatory, antidiabetic, antinociceptive, cardioprotective, neuroprotective and anticarcinogenic properties [27]. The effects of carvacrol on glioblastoma cell growth, migration, invasion and apoptosis remain unclear.

This study evaluated the anti-glioblastoma effects of the TRPM7 inhibitor carvacrol on U87 cells and investigated the potential mechanisms underlying these effects. Wound healing and transwell assays, in combination with RT-PCR, western blotting and patch-clamp recordings were used to study the effects of carvacrol.

## RESULTS

### TRPM7 mRNA and protein expression is greater in U87 cells compared to NHA cells

**Figure 1:**
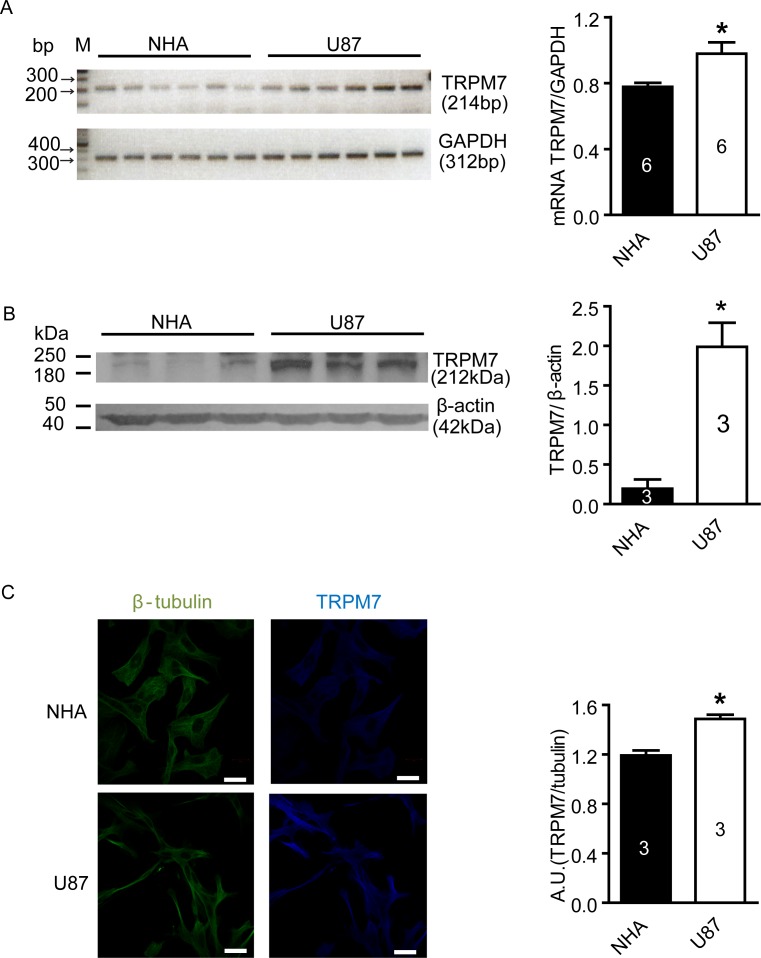
Increased expression of TRPM7 mRNA and protein in U87 cells compared to NHA cells **A**, TRPM7 mRNA in NHA and U87 cells was detected by RT-PCR. TRPM7 mRNA expression was normalized to GAPDH. The results showed that TRPM7 mRNA level in U87 cells increased compared to NHA cells (*, p<0.05, Student's *t*-test, n=6). **B**, TRPM7 protein expression in NHA and U87 cells was measured by western blotting from three different passages. After normalized to β-actin, the results showed that TRPM7 protein expression in U87 cells was higher than in NHA cells (*, p<0.05, Student's *t*-test, n=3). **C**, TRPM7 protein in situ expression in NHA and U87 cell was detected by immunofluorescence. Images were captured by a laser scanning confocal microscope and representative images are shown. The fluorescence intensity of TRPM7 staining was normalized to β-tubulin. 150 cells were chosen randomly from each experiment for analysis. Analysis showed that TRPM7 protein expression in U87 cells was more abundant than in NHA cells (white scale bar = 20 nm, *, p<0.05, Student's *t*-test, n=3).

To examine the levels of TRPM7 mRNA and protein in U87 cells, RT-PCR and western blotting were carried out, respectively. The NHA cell line was used as a control. Figure 1A shows that the amount of TRPM7 mRNA in U87 cells (normalized to GAPDH: 0.98±0.7 arbitrary unit, n=6) was significantly higher than in NHA cells (0.78±0.2, n=6. p<0.05). Western blotting demonstrated that TRPM7 protein level (normalized to β-actin) was also higher in U87 cells (1.99±0.31 arbitrary unit, n=3) compared to NHA cells (0.20±0.12, n = 3. p<0.05) (Figure 1B). TRPM7 protein levels were further measured in U87 cells using immunofluorescent staining. As shown in Figure 1C, a higher level of TRPM7 protein was observed in U87 cells compared to NHA cells in cell culture. The fluorescence intensity in U87 cells was 25% higher than NHA cells (p<0.05, n=3). Our results provide the first evidence that TRPM7 channels are highly expressed in U87 cells.

### Carvacrol inhibits TRPM7 and TRPM7-like currents in U87 cells

**Figure 2:**
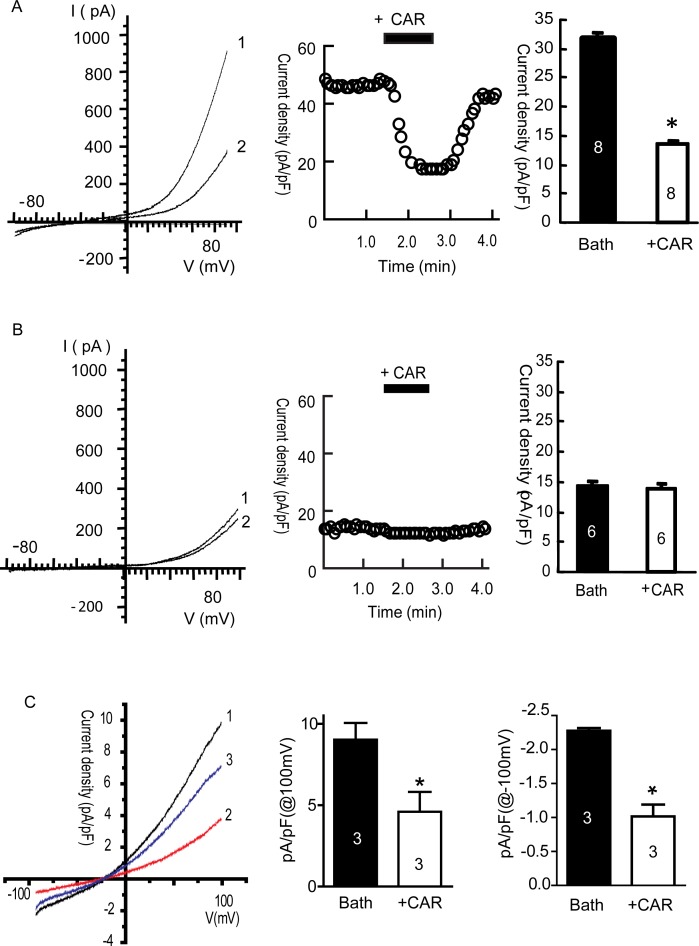
Carvacrol (CAR) blocked TRPM7 currents in HEK293 cells overexpressing TRPM7 and TRPM7-like currents in U87 cells All currents were recorded as described in the methods section. **A**, Carvacrol blocked TRPM7 currents in tetracycline (Tet)-induced TRPM7-overexpressing HEK293 cells. Left panel: representative I-V trace (1 is bath solution, 2 is 300μM carvacrol perfusion). Middle panel: representative time course of the outward current of TRPM7 at +80 mV. Right panel: analysis of outward current at +80 mV comparing perfusion with bath solution to carvacrol perfusion. TRPM7 currents were blocked by carvacrol and restored after washout of carvacrol (*, p<0.05, Student's *t*-test, n=8 cells). **B**, Carvacrol did not significantly affect background TRPM7 currents in HEK293 cells without tetracycline treatment. Left: representative current–voltage (I-V) trace of TRPM7 current (1 is bath solution, 2 is 300 μM carvacrol perfusion). Middle panel: typical time course of outward currents of TRPM7 at +80 mV. Right panel: analysis of TRPM7 outward current in bath solution and CAR perfusion. These data indicate that carvacrol perfusion does not have significant effects on background TRPM7 currents in HEK293 cells without tetracycline treatment (p>0.05, Student's *t*-test, n=6 cells). **C**, carvacrol blocks TRPM7-like currents in U87 cells. Left panel is the representative I-V trace (1 is trace of bath solution, 2 is trace of perfusion with 500 μΜ carvacrol, 3 is trace of washout of carvacrol). Middle and right panels: outward and inward currents of TRPM7 at +100mV and −100 mV. Carvacrol (500 μM) significantly blocked TRPM7-like currents in U87 cells (*, p<0.05, Student's *t*-test, n=3 cells).

To confirm the blocking effect of carvacrol on TRPM7 current, we first carried out whole-cell patch-clamp recording on HEK293 cells overexpressing recombinant TRPM7 channels. Tetracycline was used to induce TRPM7 overexpression in HEK293 cells. Figure 2A and 2B show that the TRPM7 currents in HEK293 cells treated by tetracycline were large and outwardly rectifying, whereas those in cells without tetracycline incubation were smaller and outwardly rectifying. Carvacrol (300 μM) significantly reduced TRPM7 current intensity at +90 mV in HEK293 cells treated with tetracycline, reducing TRPM7 current density by approximately 56% (Figure 2A, n = 8, p<0.05). Carvacrol did not reduce TRPM7 current intensity at +90 mV in HEK293 cells without tetracycline treatment (Figure 2A, n=6). We next tested whether carvacrol reduced TRPM7-like currents in U87 cells. As shown in Figure 2C, the intensity of outwardly rectifying current in U87 cells recorded at a current density of 100 mV was 9.0±1.0 pA/pF. Carvacrol (500μM) perfusion significantly reduced the outward current to 4.6±1.2 pA/pF (p<0.05, n=3 cells). Inward current was similarly reduced by carvacrol (p<0.05, n=3 cells). After washout of carvacrol, the inhibitory effects of carvacrol were eliminated. Our results suggest that TRPM7 channels are functional in U87 cells and channel activity is sensitive to carvacrol block.

### Carvacrol reduces U87 cell viability and proliferation

**Figure 3:**
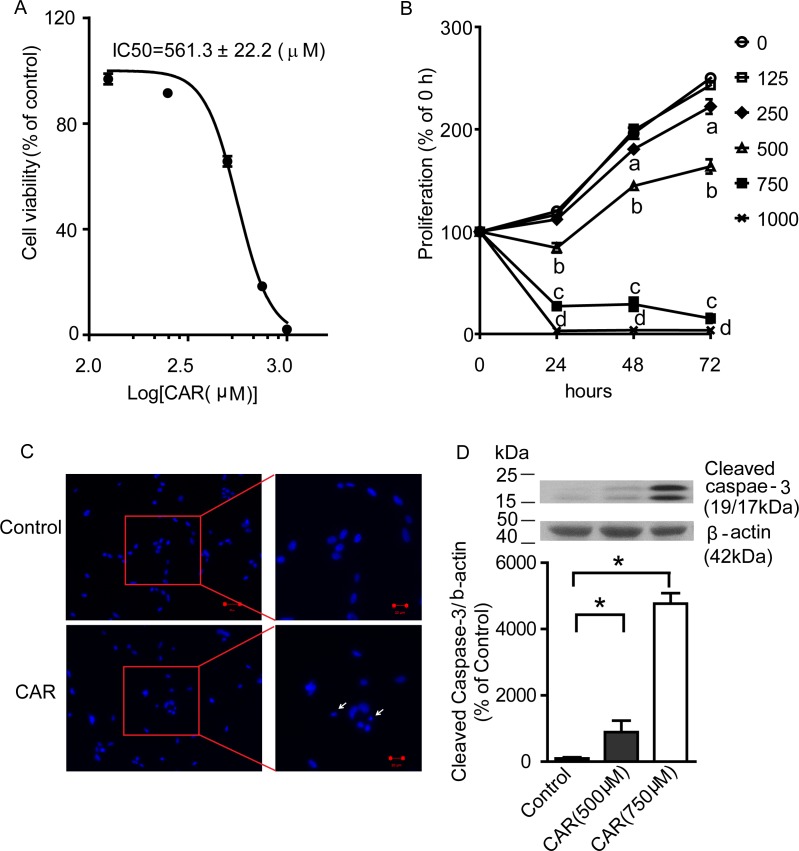
Carvacrol reduced U87 cell viability and proliferation, and induced apoptosis **A**, Carvacrol dose-dependently reduced the viability of U87 cells. U87 cells were treated with carvacrol from 125 to 1000 μM for 24 hours. An MTT assay was used to evaluate the cell viability and IC50 was calculated (n=8). **B**, carvacrol inhibited cell proliferation of U87 cells. U87 cells were treated with carvacrol (125-1000 μM) for 24, 48 and 72 hours and then an MTT assay was used to measure the proliferation. Carvacrol (250μM) significantly inhibited U87 cell proliferation at 48 and 72 hours (“a” indicates p<0.05 versus vehicle group, one-way ANOVA with subsequent Newman-Keuls test, n=8). Carvacrol (500-1000μM) significantly inhibited U87 cell proliferation at 24, 48 and 72 hours (“b,c,d” indicate p<0.05 versus vehicle group, one-way ANOVA with subsequent Newman-Keuls test, n=8). **C**, carvacrol induced U87 cell nuclear condensation. DAPI staining was used to observe nuclear condensation (arrows) as amorphological alteration indicating apoptosis. Representative images are from three independent experiments. **D**, carvacrol increased cleaved caspase-3 protein level. Western blotting was used to detect cleaved caspase-3 levels in U87 cells. Carvacrol treatment for 24 hours dose-dependently and significantly increased cleaved caspase-3 protein level in U87 cells (*, versus control, p<0.05, one-way ANOVA with subsequent Newman-Keuls test, n=6).

After verifying that carvacrol reduced TRPM7-like current activity in U87 cells, we investigated the effects of carvacrol on viability and proliferation of U87 cells using an MTT assay. Figure 3A shows that carvacrol treatment for 24 hours reduced the viability of U87 cells in a dose-dependent manner, with an IC_50_ of 561.3±22.2μM. The time course of cell proliferation is shown in Figure 3B. The proliferation of cells in the control group increased with time in culture (119.9±2.0%, 196.1±5.4% and 250.1±2.7% at 24, 48 and 72 hours). Compared with control (0.1% DMSO in culture medium), 125 μM carvacrol did not affect the proliferation of U87 cells (p>0.05, n=8). After treatment with 250 μM carvacrol, cell proliferation increased to 112.0±1.7%, 180.5±3.5% and 222.3±7.1% at 24, 48 and 72 hours, respectively. This is significantly lower than the proliferation of control cells at 48 and 72 hours (p<0.05, n=8). Carvacrol (500-1000μM) significantly inhibited proliferation of U87 cells at 24, 48 and 72 hours (Figure 3B, data presented as the mean± SEM, p<0.05, n=8 experiments).

### Carvacrol induces apoptosis in U87 cells

We next addressed whether carvacrol reduces the viability of U87 cells by enhancing apoptosis. We first measured apoptotic nuclear condensation using DAPI staining. Figure 3C shows induced apoptotic nuclear condensation after treatment with carvacrol (750 μM) for 24 hours (Figure 3C). We then measured the level of cleaved caspase-3, an active form of apoptosis-related cysteine peptidase using western blotting analysis. As shown in Figure 3D, carvacrol (500 and 750 μM) treatment for 24 hours enhanced cleaved caspase-3 levels in a dose-dependent manner. Cleaved caspase-3 levels were about 9 times higher in the 500 μM carvacrol group and 48 times higher in the 750 μM carvacrol group compared to the control group (Figure 3D, p<0.05, n=6).

### Carvacrol reduces U87 cell migration and invasion

**Figure 4:**
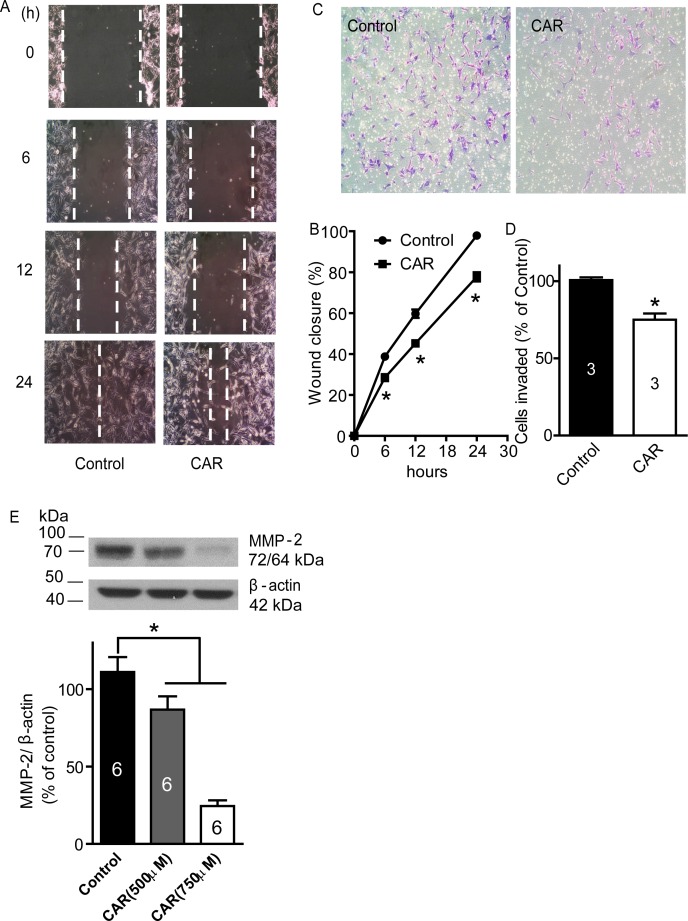
Carvacrol inhibited U87 cell migration, invasion and MMP-2 protein expression **A**, carvacrol inhibited U87 migration. The representative images of wound healing were displayed. After being scratched with a 200μL pipette tip, U87 cells were treated with CAR (500 μM) or vehicle (0.1% DMSO), then images were captured at 0, 6, 12, and 24 hours, and gap closure was analyzed **B**. The wound closure of carvacrol treatment groups at 6, 12 and 24 hours was significantly different compared to the control group at the corresponding time-point (*, p<0.05, Student's *t*-test, n=4). **C**, carvacrol inhibited U87 cell invasion. Representative images are from transwell experiments to detect cell invasion *in vitro*. **D**, analysis of transwell experiments (* versus control, p<0.05, Student's *t*-test, n=3). **E**, carvacrol dose-dependently reduced MMP-2 protein expression in U87 cells. U87 cells were treated with carvacrol (500 and 750 μM) for 24 hours. Western blotting was carried out to detect MMP-2 protein expression and β-actin was used as a loading control (* versus control, p<0.05, one-way ANOVA with subsequent Newman-Keuls test, n=6).

A wound healing assay and transwell assay were carried out to evaluate whether carvacrol alters the cell migration and invasion properties of U87 cells. Wound healing assays are used for analysis of cell migration *in vitro* [28]. In Figure 4A and 4B, cell culture images were captured at 0, 6, 12 and 24 hours after carvacrol (500 μM) treatment and the wound gap was analyzed. At 6, 12 and 24 hours, wound closure in the control group was 38.8±0.3%, 59.7±2.0% and 97.9±0.6%, which was faster than wound closure in the carvacrol group at the same time-points: 28.5±1.7%, 45.2±1.1% and 77.7±2.2% (p<0.05, n=4), respectively. This indicated that the migration of U87 cells in the carvacrol group was significantly reduced compared to the control group. In Figure 4C and 4D, transwell assay results indicated that carvacrol (500 μM) treatment significantly inhibited U87 cell invasion (75±4 invading cells in the carvacrol group versus 101±2 invading cells in the control group, p<0.05, n=3).

### Carvacrol reduces MMP-2 protein expression

The inhibition of MMP-2 activity or downregulation of MMP-2 protein levels inhibits migration and invasion of glioblastoma cell lines *in vitro* and *in vivo* [3, 4, 7]. To determine whether carvacrol exerts its effects via an MMP-2-dependent mechanism, we measured the protein level of MMP-2. Western blotting analysis showed that carvacrol (500 and 750 μM) treatment for 24 hours reduced MMP-2 protein level in U87 cells Figure 4E (86.8±8.5% in the 500 μM carvacrol group, 24.5±3.7% in the 750 μM carvacrol group, versus 111.1±9.6% in the control group, n=6, p<0.05).

### Carvacrol upregulates phosphorylation of cofilin (p-cofilin) and reduces polymerization of F-actin

**Figure 5:**
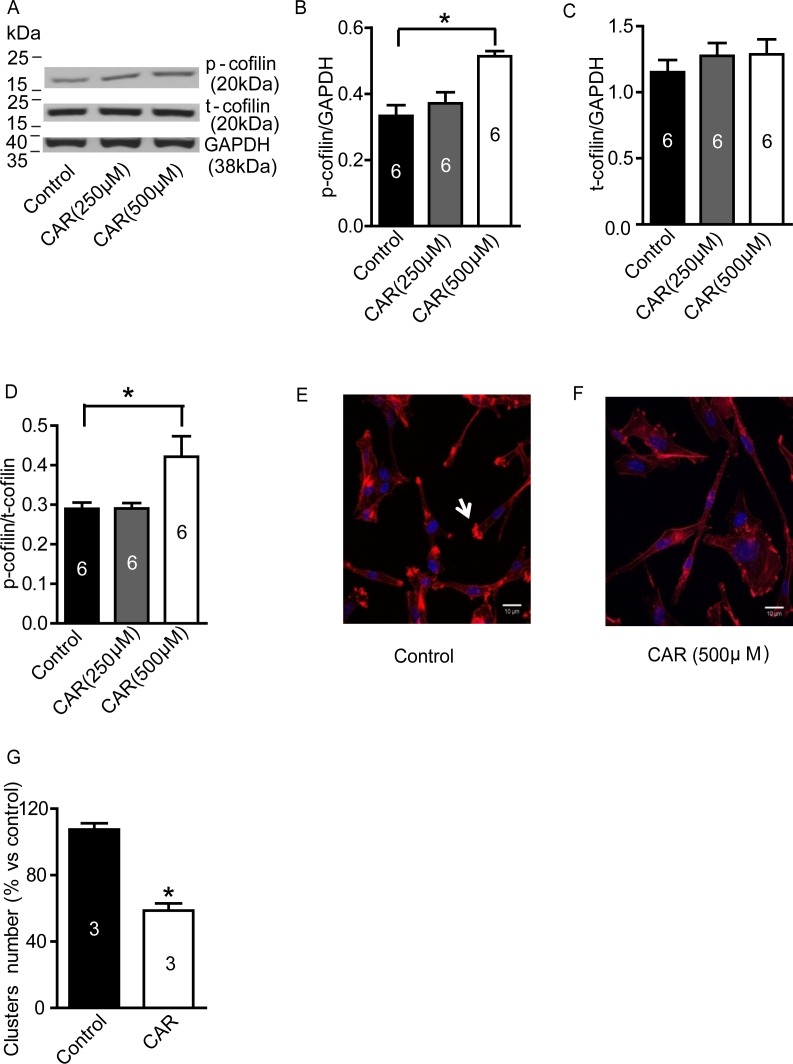
Carvacrol increased p-cofilin level and reduced F-actin polymerization in U87 cells **A-D**, Western blotting results of p-cofilin and t-cofilin protein expression in U87 cells. It showed that carvacrol (500μM) treatment for 24 hours increased p-cofilin level but not t-cofilin expression in U87 cells (*, versus control, p<0.05, one-way ANOVA with subsequent Newman-Keuls test, n=6). **E** and **F,** representative images from rhodamine phalloidin staining. U87 cells without carvacrol treatment displayed abundant bright actin clusters as indicated by arrows. **G**, analysis of the average number of bright actin clusters. The number of F-actin-rich bright actin clusters in U87 cells were significantly reduced by carvacrol (500 μM) treatment for 24 hours (* versus control, p<0.05, Student's *t*-test, n=3).

Cofilin is an actin-binding protein and depolymerizes actin filaments [29]. It is inactivated by phosphorylation [30]. We wondered whether the effects of carvacrol were mediated through regulation of cofilin. In Figure 5A-5D, western blotting analysis showed that carvacrol (500 μM) treatment for 24 hours increased the level of p-cofilin, while the total cofilin (t-cofilin) did not change. The ratio of p-cofilin/t-cofilin was significantly higher in carvacrol-treated cultures (500 μM, 0.42±0.05, n = 6) than in control cultures (0.29±0.02, n=6; p<0.05, Figure 5B). Moreover, we evaluated the effects of carvacrol on the regulation of the actin cytoskeleton in U87 cells. In Figure 5E and F, rhodamine phalloidin staining shows that cells in the control group are rich in bright actin clusters (indicated by arrow), while cells treated with carvacrol- for 24 hours have fewer of these clusters (Figure 5G).

### Carvacrol suppresses PI3K/Akt and MEK/MAPK signaling pathways

**Figure 6:**
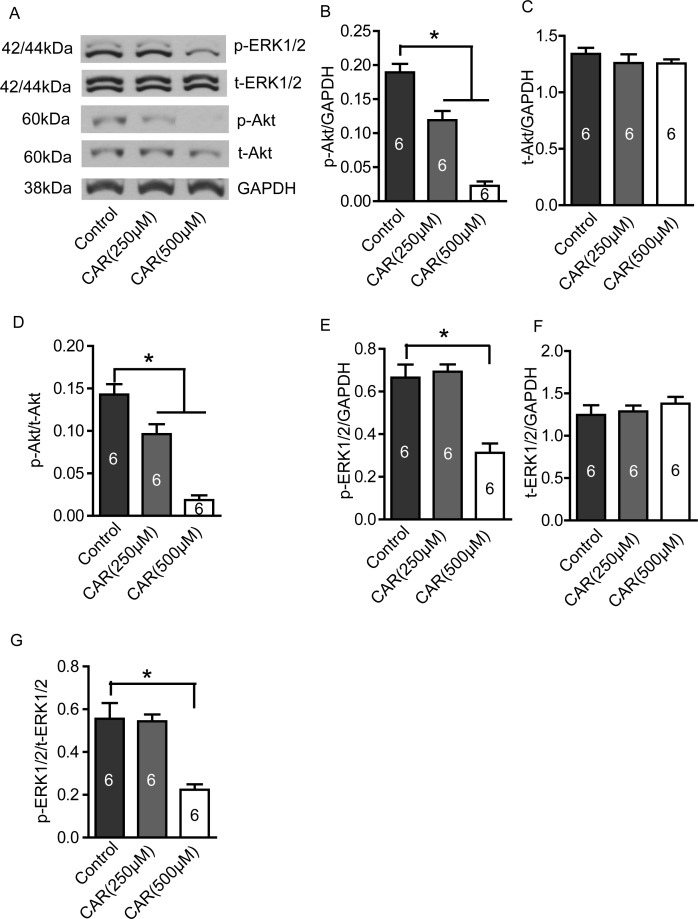
Carvacrol reduced p-Akt and p-ERK1/2 protein levels in U87 cells U87 cells were treated with carvacrol (250 and 500 μM) for 24 hours, and then protein expression was detected by western blotting. **A**, Representative images of western blotting results. **B**, Carvacrol (250 and 500 μM) significantly reduced p-Akt protein level in a dose-dependent manner. * p<0.05, one-way ANOVA with subsequent Newman-Keuls test, n=6. **C**, Carvacrol did not significantly affect t-Akt protein expression (p>0.05, n=6). **D**, Ratio of p-Akt/t-Akt decreased in the carvacrol (250 and 500 μM) group in a dose-dependent manner. *, p<0.05, one-way ANOVA with subsequent Newman-Keuls test, n=6. **E**, Carvacrol (500μM) significantly decreased p-ERK1/2 protein level. * p<0.05, one-way ANOVA with subsequent Newman-Keuls test, n=6. **F**, Carvacrol did not significantly affect t-ERK1/2 protein expression (p>0.05, n=6). **G**, Ratio of p-ERK1/2/t-ERK1/2 decreased in the carvacrol (500 μM) group. *, p<0.05, one-way ANOVA with subsequent Newman-Keuls test, n=6.

PI3K/Akt and MEK/MAPK signaling pathways are involved in the regulation of proliferation, migration and invasion of glioblastoma cells. As shown in Figure 6A, both p-Akt and p-ERK1/2 protein levels were reduced in carvacrol-treated (500 μM) cells. Densitometry analysis shows that p-Akt levels, when normalized to GAPDH levels, are significantly reduced in the carvacrol group (Figure 6B, carvacrol (250 μM): 0.12±0.01; carvacrol (500 μM): 0.02±0.01; * p<0.05, n=6) compared to the control group (0.19±0.01). Carvacrol treatment for 24 hours did not significantly affect total Akt (t-Akt) protein expression in U87 cells (Figure 6C, p>0.05, n=6). In addition, the ratio of p-Akt/t-Akt in the carvacrol group was smaller than in the control group (Figure 6D, carvacrol (250 μM): 0.10±0.01; carvacrol (500 μM): 0.02±0.005; control group: 0.14±0.01; all normalized to GAPDH; * p<0.05, n=6). In addition, carvacrol (500 μM) treatment for 24 hours reduced p-ERK1/2 levels (Figure 6E, 500μM carvacrol: 0.31±0.04% versus control: 0.66±0.06 normalized to GAPDH, * p<0.05, n=6), and p-ERK1/2-t-ERK1/2 ratio (Figure 6G, 500 μM carvacrol: 0.22±0.03% versus control group: 0.56±0.07 normalized to GAPDH, * p<0.05, n=6). The total ERK1/2 protein expression did not change significantly (Figure 6F, p>0.05, n=6).

### TRPM7 silencing reduces U87 cell viability, migration and invasion

**Figure 7:**
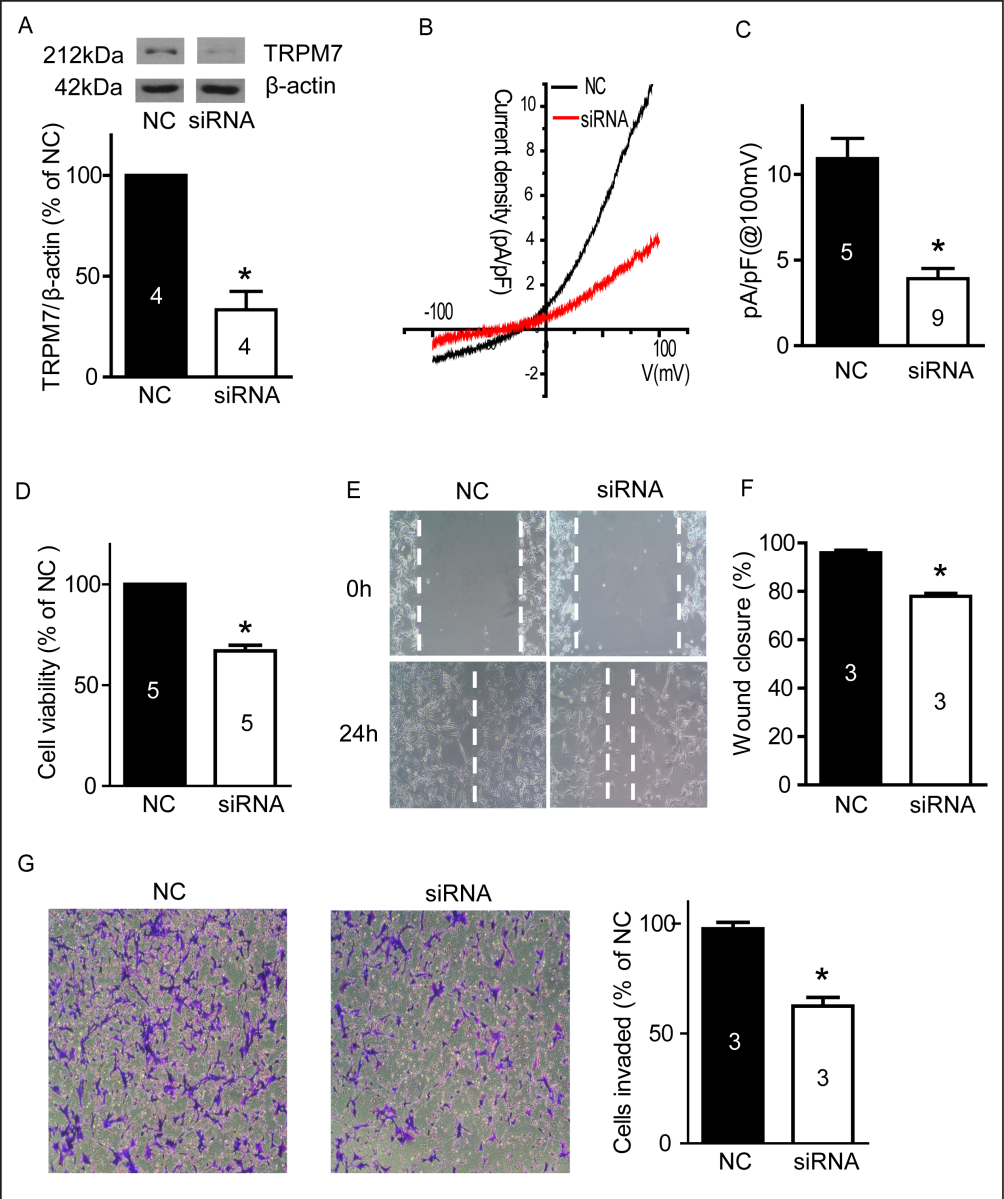
Silencing TRPM7 reduced cell viability, migration and invasion **A**, U87 cells were transfected with siRNA for 72 hours, then western blotting was carried out. TRPM7 siRNA significantly decreased TRPM7 protein expression. *, p<0.05, Student's *t*-test, n=4. **B**, Whole-cell patch-clamp experiments were performed after siRNA transfection for 72 hours. It showed the representative current–voltage (I-V) traces of TRPM7-like current in U87 cells with NC and siRNA transfection. **C**, TRPM7-like currents in U87 cells were significantly inhibited by TRPM7 siRNA. * versus NC, p<0.05, Student's *t*-test, from 5 cells in NC group and 9 cells in siRNA group. **D**, An MTT assay shows that TRPM7 silencing significantly reduced U87 cell viability. *, p<0.05, Student's *t*-test, n=5. **E** and **F**, Wound healing experiments show that TRPM7 silencing significantly inhibited U87 cell migration. *, p<0.05, Student's *t*-test, n=3. **G**, Transwell experiments show that TRPM7 silencing significantly reduced cell invasion. *, p<0.05, Student's *t*-test, n=3.

Silencing of TRPM7 has been previously shown to reduce cell viability, migration, and invasion of A172 cells- a human glioma cell line [31]. In this study, we observed the effects of silencing TRPM7 on these functions in U87 cells. As shown in Figure 7A, TRPM7 siRNA significantly decreased TRPM7 protein by ~70% (p<0.05, n=4). We further employed whole-cell patch-clamp recording to measure TRPM7-like current in U87 cells. TRPM7 siRNA transfection of 72 hours significantly reduced TRPM7-like current in U87 cells (Figure 7B and 7C, p<0.05, n=5 cells in NC group and 9 cells in siRNA group). These data suggests that TRPM7 siRNA can effectively silence TRPM7 by reducing protein expression and ion channel activity. As shown in Figure 7D, silencing TRPM7 significantly reduced cell viability (67.0±2.8% of NC, p<0.05, n=5). Moreover, silencing TRPM7 significantly inhibited U87 cell migration (Figure 7E and 7F, siRNA: 77.9±1.3% versus NC: 95.9±1.1% wound closure, p<0.05, n=3) and invasion (Figure 7G, S2: 62.5±4.0% of NC, p<0.05, n=3).

### Silencing of TRPM7 suppresses PI3K/Akt and MEK/MAPK signaling pathways and reduces MMP-2 protein expression

**Figure 8:**
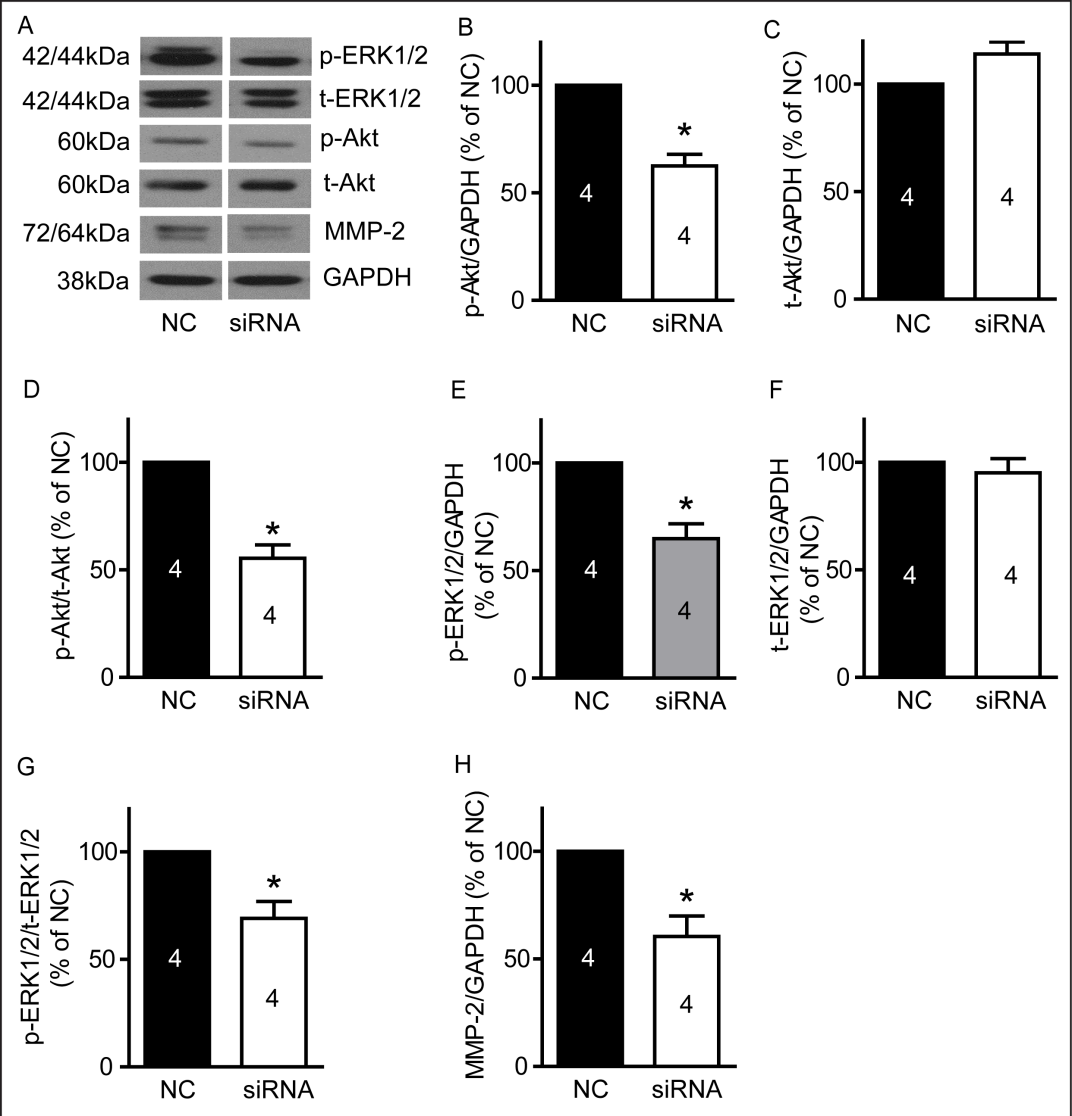
TRPM7 silencing reduced p-Akt, p-ERK1/2 and MMP-2 protein levels in U87 cells U87 cells were transfected with TRPM7 siRNA for 72 hours, and then protein expression was detected by western blotting. **A**, Representative images of western blots (n=4). **B**, TRPM7 silencing significantly decreased p-Akt protein level. *, p<0.05, Student's *t*-test, n=4. **C**, TRPM7 silencing did not significantly affect t-Akt protein expression (p>0.05, n=4). **D**, The ratio of p-Akt/t-Akt is reduced in the TRPM7-silenced group. *, p<0.05, Student's *t*-test, n=4. **E**, TRPM7 silencing significantly decreases p-ERK1/2 protein level. *, p<0.05, Student's *t*-test, n=4. **F**, TRPM7 silencing did not significantly regulate t-ERK1/2 protein expression (p>0.05, n=4). **G**, Ratio of p-ERK1/2/t-ERK1/2 is reduced in the TRPM7 silencing group. *, p<0.05, Student's *t*-test, n=4. **H**, TRPM7 silencing significantly decreased MMP-2 protein expression. *, p<0.05, Student's *t*-test, n=4.

We examined p-Akt/t-Akt, p-ERK1/2/t-ERK1/2 and MMP-2 protein expression in U87 cells after silencing TRPM7. As show in Figure 8, silencing TRPM7 significantly reduces p-Akt (Figure 8B, 62.6±5.4% of NC, p<0.05, n=4) and p-ERK1/2 (Figure 8E, 64.8±6.9% of NC, p<0.05, n=4) protein levels, as well as the ratio of p-Akt/t-Akt (Figure 8D, 55.5±6.2% of NC, p<0.05, n=4) and p-ERK1/2/t-ERK1/2 (Figure 8G, 69.1±7.9% of NC, p<0.05, n=4) while it did not significantly affect t-Akt and t-ERK1/2 protein expression (Figure 8C and 8F, p>0.05, n=4). Moreover, silencing TRPM7 reduced MMP-2 protein expression (Figure 8H, 60.4±9.6% of NC, p<0.05, n=4).

## DISCUSSION

In this study, we demonstrated that TRPM7 was upregulated in U87 cells. Carvacrol treatment reduced cell viability, proliferation, migration and invasion of U87 cells, and induced apoptosis by blocking TRPM7. Moreover, we showed that carvacrol decreased matrix metalloproteinase-2 (MMP-2) protein levels and increased levels of phosphorylated cofilin in U87 cells, which may be the underlying mechanism of the inhibitory effects of carvacrol on U87 cell migration and invasion. Carvacrol treatment also inhibited PI3K/Akt and MEK/MAPK signaling pathways. In the parallel experiments, silencing TRPM7 elicited effects on U87 cellsconsistent with those of carvacrol, including the inhibition of cell viability, migration and invasion, the attenuation of PI3K/Akt and MEK/MAPK signaling pathways, and the reduction of MMP-2 protein expression.

Tumorigenesis is a product of the imbalance between cell proliferation and apoptosis. However, the process of tumor growth is complicated and does not simply result from enhanced cell proliferation and/or reduced apoptosis. The fact that significant cell death occurs in tumors explains why the amount of cell proliferation does not always correlate with tumor growth rate [32]. Standard care for glioblastomas includes surgery, chemotherapy (temozolomide (TMZ), a DNA alkylating agent) and radiation therapy [33]. However, malignant glioma cells have intense resistance to death-inducing stimuli such as radiotherapy and chemotherapy. Thus, it is clinically important to discover novel therapeutic targets for the treatment of glioblastomas.

Recent studies have focused on the TRPM7 pathway in glioma cells and stem-like cells derived from human glioma cell lines [34]. Consistent with previous reports [16, 18], we found high mRNA and protein levels of TRPM7 in human U87 cells (Figure 1). Liu et al reported that TRPM7 promotes the proliferation, migration, and invasion of A172 cells, a glioma cell line [34]. TRPM7 activates JAK2/STAT3 and/or Notch signaling pathways and leads to increased cell proliferation and migration [34]. Activation of STAT3 by TRPM7 also directly regulates expression levels of ALDH1. The Notch signaling pathway has potential as a therapeutic target since it plays important roles in proliferation, differentiation, apoptosis, and cancer stem cell regulation. STAT3 is a tumor suppressor in PTEN-deficient glioblastoma tumors but has a promoting function in EGFRvIII-expressing tumors [35]. Apart from being a stem cell marker in human glioblastoma, ALDH1 may also have functional roles related to self-protection, differentiation, expansion, and proliferation [36, 37]. TRPM7 suppression by shRNAi inhibits the growth, proliferation, migration, and invasion of A172 cells [31]. Thus, TRPM7 appears to be a promising target for therapeutic intervention in glioblastoma. In this study, we investigated whether blocking TRPM7 activity using a pharmacological approach mimics the effects of TRPM7 suppression.

Carvacrol blocks TRPM7 channels with an IC_50_ of 307 μM in HEK cells over-expressing TRPM7 [20]. We have verified that carvacrol (500 μM) blocks both recombinant TRPM7 and endogenous TRPM7-like currents in U87 cells (Figure 2). Human glioblastoma cell line A172 is very similar to the human U87 cell line used in this study - both of which are human malignant astrocytoma cell lines [38]. We found that blocking TRPM7 channelswith carvacrol reduced viability, proliferation, migration and invasion of U87 cells. These results were further confirmed by silencing TRPM7, which was shown to reduce cell viability, migration and invasion. The effects of TRPM7 silencing on U87 cells is consistent with a previous report [31]. The IC_50_ of carvacrol for the reduction of cell viability and proliferation of U87 cells was 561.3 μM in our study. A lower carvacrol concentration has been reported to exert antiproliferative effects in leiomyosarcoma cells [39]. A previous study reported that carvacrol treatment at concentrations below 200 mg/L (1.33 mM) for 24 hours did not affect the viability of healthy neurons and N2a cancer cells [40]. The concentrations of carvacrol used in our study are below this concentration. Carvacrol also has non-specific channel targets such as the activation of TRPV3 and TRPA1 channels [41], and the inhibition of TRPL channels at a similar concentration [20]. To date however, there is no evidence illustrating the involvement of TRPV3 or TRPA1 in the regulation of glioma cell function. Considering that ion channels are the second largest target for drug development, it is important to study TRPM7 channels for drug development and translational research.

Carvacrol has been shown to provide protection against apoptosis in ischemia-reperfusion injury [42], acute myocardial infarction [43], thioacetamide-induced hepatotoxicity [44], and H_2_O_2_ induced injury in isolated pancreas islet cells [45]. However, carvacrol exhibits pro-apoptotic effects in multiple cancer cell lines, including DBTRG-05MG human glioblastoma cells [46], a breast cancer cell line [47] and a human hepatocellular carcinoma cell line HepG-2 [48], via either ROS generation [46] or mitochondrially mediated apoptosis [48]. In this study we show that carvacrol triggers apoptosis-related processes in U87 cells, supporting the notion that the effects of carvacrol are cell type-dependent.

Cell migration is a complex mechanism essential for biological processes such as immune response, wound healing, tissue repair and embryogenesis. Errors in cell migration lead to a variety of pathologies, including cancer invasion and metastasis [49, 50]. Cell migration is driven by the formation of specific cytoskeletal structures known as lamellipodia, filopodia and invadosomes, which initiate the protrusion of the cell membrane [51]. Formation of these structures is in turn highly dependent on the spatial and temporal assembly and disassembly of actin filaments at the protruding edge [52]. To date, several intracellular pathways that regulate the formation of these structures have been identified and the overexpression of the proteins involved in these pathways has been shown in several types of cancers. These include the Wiskott-Aldrich syndrome protein (WASP) family (Apr2/3 complex, LIM kinase), cofilin and cotractin pathways [53]. While all of these pathways have been extensively studied in the context of cancer cell migration and invasion, the LIM kinase – cofilin pathway is considered to be essential in regulating cancer cell motility [53]. Cofilin is highly overexpressed in rat glioblastoma, human adenocarcinoma, breast cancer, ovarian cancer and pancreatic cancer cells [54-58]. The amount of phosphorylated (inactive) cofilin is significantly decreased in cell lines derived from Jurkat T-lymphoma, kidney, liver, colon and cervical cancers [58]. In particular, cofilin has been shown to be involved in the formation of invadopodia [59] (the major structure involved in cancer cell motility), and inhibiting confilin with siRNA or overexpression of the LIM kinase active domain reduces the invasion of carcinoma cells [60, 61]. Cofilin acts through severing cross-linked actin filaments, thus increasing the number of free-pointed and barbed ends, as well as the number of actin monomers available for polymerization and formation of invadopodial protrusions [52]. In our study, carvacrol enhances p-cofilin levels without affecting total cofilin levels, which subsequently attenuates U87 cells migration and invasion. Invadosomes were observed in F-actin-rich areas at the leading edge of the cell membrane [62]. In accordance with our findings, TRPM7 inhibition also causes a reduction in invadosome formation in N1E-115 neuroblastoma cells [62]. Immunofluorescent images showed that untreated cells possess actin-rich invadosomes that are visualized by rhodamine phalloidin (Figure 5E). In contrast, cells treated with 500 μM carvacrol exhibit longer, unsevered actin filaments and fewer actin-rich hotspots at the leading edge of the cells. Matrix metalloproteinase-2 (MMP2) is highly localized to the invadosomes of cancer cells, causing degradation of the extracellular matrix and aiding in the invasion process [63]. Carvacrol decreased MMP2 levels along with invadosome number - thus reducing the ability of U87 cells to migrate and metastasize.

PI3K/Akt and MEK/MAPK signaling pathways are considered to play a critical role in proliferation, migration and invasion of glioblastoma cells. Inhibitors that target these signaling pathways are currently under evaluation in clinical trials [7]. However, majority of patients fail to respond to treatments that suppress only one of these signaling pathways [64] (likely due to cross-talk between these pathways). Therefore, blocking both of these signaling pathways simultaneously may be a rational and more effective strategy in the treatment of glioblastoma [65]. Several studies suggest that TRPM7 regulates PI3K/Akt and MEK/MAPK pathways. Down-regulation of TRPM7 decreases the level of p-Akt in OVCA cells and human lung fibroblasts, and also decreases the level of p-ERK1/2 in breast cancer cells [66-68]. Moreover, silencing TRPM7 in hepatic stellate cells prevents an increasing in p-Akt and p-ERK1/2 levels induced by PDGF-BB [69]. In this study, we found that both carvacrol and the silencing of TRPM7 reduced p-Akt and p-ERK1/2 protein levels, suggesting that blocking TRPM7 by carvacrol inhibits PI3K/Akt and MEK/MAPK signaling pathways. The molecular mechanism by which TRPM7 interacts with these signaling pathways remains unclear. PI3K/Akt and MEK/MAPK signaling are regulated by phosphoinositide-specific phospholipase C (PLC) [70], and several phospholipase C (PLC) isozymes also interact with the TRPM7 α-type serine/threonine protein kinase domain [71]. Hence, we speculate that TRPM7 likely regulates PI3K/Akt and MEK/ERK signaling through an interaction with PLC (A schematic diagram is shown in Figure 7). However, further study is needed to determine if this is indeed the case.

In conclusion, carvacrol reduced cell viability, proliferation, migration and invasion in the human glioblastoma U87 cell line, as well as induced apoptosis - likely by inhibiting TRPM7 activity and both the PI3K/Akt and MEK/MAPK signaling pathways. Carvacrol may regulate U87 cell migration and invasion through the reduction of MMP-2 protein expression and an increase in p-cofilin levels. Our findings suggest that carvacrol has therapeutic potential for the treatment of glioblastomas.

**Figure 9:**
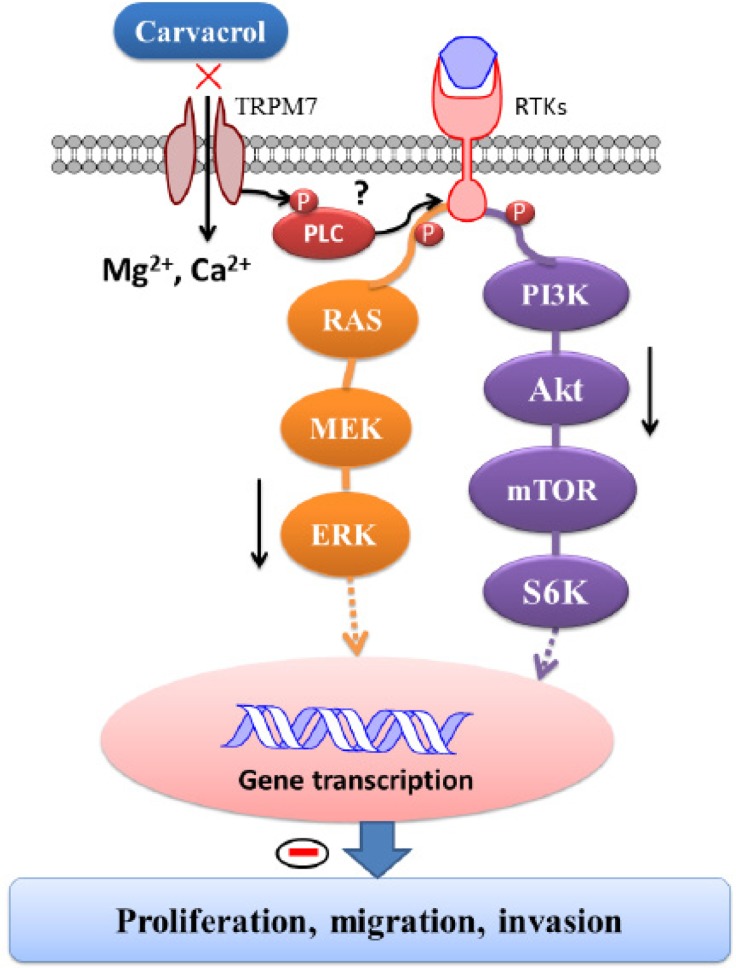
Schematic diagram of signaling mechanisms involved in the effects of carvacrol on proliferation, migration and invasion of U87 cells TRPM7 is constitutively active in resting cells. When exposed to various extracellular or cytosolic stress or stimuli, TRPM7 regulates entry of Mg^2+^ and Ca^2+^. Moreover, TRPM7 has an α-type serine/threonine protein kinase domain that can phosphorylate itself, as well as cytosolic substrates such as modulating phosphorylation of PLC and subsequently regulating PI3K/Akt and MEK/MAPK signaling pathways, leading to functional gene transcription and translation. As a consequence, the TRPM7 channel modulates cellular proliferation, migration and invasion. It indicates that carvacrol exerts its anti-glioblastoma effects by inhibiting TRPM7 and thus PI3K/Akt and MEK/MAPK signaling pathways.

## MATERIALS AND METHODS

### Reagents

The SuperScript^®^ III First-Strand Synthesis System and AccuPrime™ Taq DNA Polymerase System were purchased from Invitrogen/Life Technologies Corporation, USA. Anti-TRPM7 (cat#ab85016), anti-Cofilin (phospho S3) antibody (cat#ab12866) and anti-Cofilin antibody [EP6376] (cat#ab134963) were purchased from Abcam, USA. Diamidino-2-phenylindole (DAPI, cat#4083), MMP-2 (D8N9Y) rabbit mAb (cat#13132), cleaved caspase-3 (Asp175) antibody (cat#9661), phosphor-Akt (ser473) antibody (p-Akt, cat#9271), Akt antibody (t-Akt, cat#9272) and phospho-p44/42 MAPK antibody (p-ERK1/2, cat#5726) were purchased from Cell Signaling Technology, USA. Anti-MAP Kinase ERK1/ERK2 Rabbit pAb (t-ERK1/2, cat#442704) was purchased from Millipore. Pierce™ BCA Protein Assay Kit was product of Pierce Biotechnology, USA. All cell culture materials were purchased from Gibco Life Technologies Corporation (USA). Sodium Dodecyl Sulfate (SDS), sodium chloride (NaCl) and phenylmethylsulfonyl fluoride (PMSF) were purchased from Bioshop, Canada. All other reagents used were obtained from Sigma-Aldrich, USA unless mentioned otherwise.

### Cell culture

Human glioblastoma cell line U87 was received from the American Type Culture Collection (Manassas, VA) and normal human astrocytes (NHA, Clonetics, East Rutherford, NJ) were plated on 10-cm culture dishes and cultured in complete DMEM (Gibco, USA) supplemented with 10% heat-inactivated fetal bovine serum (FBS), 100 U/mL penicillin and streptomycin (Gibco, USA) in 5% CO_2_ and 95% humidified air atmosphere at 37ºC. HEK-293 cells with stable expression of Flag-murine TRPM7/pCDNA4 were cultured with MEM supplemented with 10% FBS, blasticidin (5 μg/mL, Sigma-Aldrich, USA), Glutamax-1 (2mM, Invitrogen, USA) and zeocin (0.4 mg/mL, Invitrogen, USA). TRPM7 expression was induced by adding 1 μg/mL tetracycline (Sigma-Aldrich, USA) to the culture.

### RT-PCR

Total RNA was isolated with Trizol reagent (Invitrogen, USA) according to the manufacturer's instructions. First-strand cDNA synthesis and PCR reactions were also carried out according to the product directions. PCR amplifications were carried out with the following primers: TRPM7 (F:5′-CTTATGAAGAGGCAGGTCATGG-3′, R:5′-CATCTTGTCTGAAGGACTG-3′, product size is 214 bp). GAPDH (F: 5′-AATCCCATCACCATCTTCC-3′, R: 5′-AGTCCTTCCACGATACCAA-3′, product size is 312 bp). PCR reaction conditions: denaturing at 94ºC for 30 seconds, annealing at 50ºC for 30 seconds, and polymerization at 72ºC for 5 min. PCR products were electrophoresed on a 2% agarose gel containing ethidium bromide. Images were captured using BIO-RAD Gel Doc 2000. Bands were analyzed using Image-Pro Plus software.

### Western blotting

Western blotting experiments were performed as previously described [72, 73]. Cells were scraped in RIPA buffer plus proteinase inhibitor cocktail (50 mM Tris, 150 mM NaCl, 1 mM EDTA, 1% Triton X-100, 0.1% SDS, 1% Sodium deoxycholate, 1 mM PMSF, 1 mM Na3VO4, 1 mM NaF, 1 μg/mL aprotinin, 1 μg/mL leupeptin, 1 μg/mL pepstatin). Protein concentration of samples was determined with the bicinchoninic acid (BCA) assay method. Equivalent amounts of sample protein were separated in 8 or 12% SDS-PAGE gel and transferred to nitrocellulose membrane (Millipore, USA) using a semi-dry transfer method (200 mA per gel, 60 min). The membrane was then blocked with 5% milk in TBS with 0.1% tween-20, and incubated with primary antibodies at 4ºC overnight as follows: anti-TRPM7 (1:1000), anti-cofilin (1:1000), anti-cofilin (phospho S3, 1:1000), anti-MMP-2 (1:1000), anti-cleaved caspase-3 (1:1000) anti-p-Akt (1:1000), anti-Akt (1:1000), anti-p-ERK1/2 (1:1000), anti-ERK1/2 (1:1000) and anti-β-actin (1:1000) antibodies, followed by incubation with anti-rabbit or anti-mouse HRP-labeled secondary antibody (1:8000) at room temperature (RT) for 1 hour. Bands were developed with a chemiluminescence reagent system (PerkinElmer Life Sciences Inc., Boston, MA). Densitometry was carried out using Image-Pro Plus software.

### Immunofluorescent staining

U87 and NHA cells (5×10^4^ cells/mL) were maintained on 18-mm round Poly-D-Lysine-coated coverslips (Warner Instruments, USA) for 24 hours, fixed with 4% paraformaldehyde for 20 minutes at RT, and then permeabilized for 20 min with 0.1% Triton X-100 in PBS. Cells were incubated overnight at 4°C with anti-TRPM7 (ab729, Abcam, 1:50) and β-tubulin (mouse mAb, Invitrogen, USA, 1:500) antibodies in 2% bovine serum albumin (BSA, Bioshop, Canada), 2% FBS and 0.2% fish gelatin (Sigma-Aldrich, USA). The cells were then incubated with Alexa-Fluor 488 conjugated anti-mouse and Alexa-Fluor 405 conjugated anti-goat (1:500, Molecular Probes, USA) antibodies for 2 hours at RT. Fluorescence was visualized with an LSM700 Zeiss confocal microscope (Carl Zeiss Inc., Gottingen, Germany).

### Rhodamine Phalloidin and DAPI fluorescent staining

Rhodamine Phalloidin staining was carried out according to the manufacturer's instructions. Cells were incubated with Rhodamine Phalloidin (1:50; Molecular Probes, USA) to label F-actin, and with DAPI (1 μg/mL) to label nucleic acid, for 15 minutes at RT. Cell images were captured from at least 6 randomly chosen areas using LSM700 Zeiss confocal microscope. Bright F-actin clusters was calculated by Image-Pro Plus software using the cell counter tool and was normalized to cell number (> 100 cells per group).

### Electrophysiology

Whole-cell patch-clamp recording was used to study TRPM7 currents from TRPM7-overexpressed HEK-293 cells and TRPM7-like currents from U87 cells using an Axopatch 700B (Axon Instruments, Inc.) [9]. Currents were recorded using a 400 ms voltage ramp protocol (−100 to +100 mV) with an interval of 5s at 2 kHz and digitized at 5 kHz. pClamp 9.2 software was used for data acquisition and Clampfit 9.2 was used for data analysis. All experiments were carried out at RT. Patch pipette resistance was between 3-5 megaohms after filling with pipette solution containing (in mM): 145 cesium methanesulfonate, 8 NaCl, 10 EGTA, and 10 HEPES, pH adjusted to 7.2 with CsOH. The bath solution contained (in mM) 140 NaCl, 5 KCl, 2 CaCl2, 20 HEPES, and 10 glucose (pH was adjusted to 7.4 with NaOH) [9]. When recording the TRPM7-like currents in U87 cells, 500 nM tetrodotoxin and 5 μM nimodipine was added to the bath solution.

### Silencing experiments

Nonspecific control siRNA (NC) and 3 pairs of siRNA for human TRPM7 (NM_017672) were purchased from Shanghai GenePharma Co., Ltd (China). The TRPM7 siRNA sequences were as follows: sense 5′-GCAGGACCUUAUGUAAUGATT-3′ and antisense 5′-UCAUUACAUAAGGUCCUGCTT-3′. The nonspecific control siRNA sequences were as follows: sense 5′-UUCUCCGAACGUGUCACGUTT-3′ and antisense 5′-ACGUGACACGUUCGGAGAATT-3′. SiRNA transfection was performed following the manufacturer's instructions using Lipofectamine RNAiMAX Reagent (Life Technologies, USA) and 120 nM siRNA. Western blotting, MTT assay, wound healing and invasion experiments were carried out after transfection with siRNA for 72 hours.

### Cell viability and proliferation assay

Cell viability was assessed by MTT assay as previously described [73]. When incubated with U87 cells in culture medium, the ratio of yellow MTT to purple formazan is reduced in the mitochondria of living cells. Absorbance at 490 nm was used to quantify the amount of MTT, which was assumed to correlate to the number of viable (living) cells. Cells seeded on 96-well culture plates at a density of 5×10^4^ cells/mL were treated with various concentrations of carvacrol (from 125 μM to 1000 μM) for 24, 48 or 72 hours. MTT reagent (0.5 mg/ml MTT in PBS) was diluted into completed medium (dilution ratio of 1:10) and added to each well. After incubating in a CO2 incubator for 3 hours, the medium was aspirated from each well and 200 μL DMSO was added. The absorbance was measured in a microplate reader (Syngery H1, Biotek, USA) at 490 nm. Cell viability was expressed as a percentage of the control (0.1% DMSO).

### Apoptotic morphological changes assay

The apoptotic morphological change was examined using DAPI staining as previously described [72, 73]. Cells were fixed with 4% paraformaldehyde for 20 minutes at RT, then incubated with DAPI (1μg/mL) for 15 minutes. At least 6 fields were randomly chosen and images were captured using ZEISS AXIO fluorescence microscope equipped with a 20× objective (Zeiss, Germany). Cells with condensed chromatin or shrunken, irregular, or fragmented nuclei were considered to be apoptotic.

### Cell migration assay

Cell migration was measured using a wound healing assay as described previously [74]. In brief, cells were seeded in 6-well plates at a density of 5×10^4^ cells/mL and grown to over 90% confluence. The monolayer of cells was scratched with a 200 μL pipette tip to create a wound gap, and treated with either carvacrol (500 μM) or vehicle control (0.1% DMSO) at various time points. Cell images were obtained with a digital camera connected to a phase-contrast Olympus microscope (CKX41, 10× objective). The same visual field was used throughout the experiment. The wound gap was measured by Image-Pro Plus software with the wound healing tool. Wound closure was calculated using the following formula: Wound closure (%) = Gap(T-T0)/GapT0*100% (where T is the treatment time and T0 is the time that the wound was induced).

### Transwell assay to evaluate cell invasion

Transwell assay experiments were carried out according to the manufacturer's instructions. BioCoat Matrigel invasion chambers (8-μm polycarbonate Nucleopore filters, Cat. 354480. BD BioSciences) were used to examine U87 cell invasion. After treatment with carvacrol (500 μM) or vehicle control (0.1% DMSO) for 24 hours, 100 μL of cells (2.5×10^4^ cells/mL) in FBS-free DMEM were added to the top chamber. 600 μL of complete medium was added in the bottom chamber as a chemoattractant. Invading cells that degrade the Matrigel and move on to the lower membrane surface in the chamber. Cells were fixed in 75% ethanol and stained with crystal violet (0.1%). Images of the invaded cells were captured with a digital camera connected to an Olympus microscope (CKX41). The number of invading cells was counted using Image-Pro Plus software with the cell counter tool.

### Statistical analysis

Data are presented as means±SEM. ANOVA with subsequent Newman-Keuls test was used to determine statistical significance for multiple comparisons, and Student's *t*-test was used to compare two groups. p<0.05 was considered statistically significant.
